# Risk Factors for Colon Cancer in Northeastern Thailand: Interaction of *MTHFR* Codon 677 and 1298 Genotypes with Environmental Factors

**DOI:** 10.2188/jea.JE20090140

**Published:** 2010-07-05

**Authors:** Supannee Sriamporn Promthet, Chamsai Pientong, Tipaya Ekalaksananan, Surapon Wiangnon, Kirati Poomphakwaen, Nopparat Songserm, Peechanika Chopjitt, Malcolm A Moore, Shinkan Tokudome

**Affiliations:** 1 Department of Epidemiology, Faculty of Public Health, Khon Kaen University, Thailand; 2 Department of Microbiology, Faculty of Medicine, Khon Kaen University, Thailand; 3 Cancer Unit, Faculty of Medicine, Khon Kaen University, Thailand; 4 Faculty of Science and Technology, Loei Rajabhat University, Thailand; 5 Department of Community Health, Faculty of Science, Ubon Ratchathani Rajabhat University, Thailand; 6 UICC Asian Regional Office for Cancer Control, Tokyo, Japan; 7 National Institute of Health and Nutrition, Tokyo, Japan

**Keywords:** colon cancer, *MTHFR*, polymorphism, environmental factors, Thailand

## Abstract

**Background:**

Polymorphisms in methylenetetrahydrofolate reductase (MTHFR), such as *MTHFR* C677T and A1298C, are associated with several cancers. This study aimed to evaluate the effects of *MTHFR* polymorphisms on colon cancer risk and possible interactions with environmental factors in a population from northeastern Thailand.

**Methods:**

This hospital-based case–control study was conducted during 2002–2006; 130 colon cancer cases and 130 age- and sex-matched controls were enrolled. Information was collected and blood samples were obtained for assay of *MTHFR* C677T and A1298C polymorphisms by polymerase chain reaction with restriction fragment length polymorphism techniques. Associations between variables of interest and colon cancer were assessed using conditional logistic regression.

**Results:**

Increased risk of colon cancer was associated with alcohol consumption and bowel habits. Alcohol drinkers who consumed ≤0.50 or >0.50 units of alcohol per day had elevated risks (OR_adj_ = 3.5; 95% CI: 1.19–10.25 and OR_adj_ = 1.71; 95% CI: 0.74–3.96, respectively). The risk was also higher in subjects with frequent constipation (11.69; 2.18–62.79) and occasional constipation (3.43; 1.72–6.82). An interaction was observed between the *MTHFR* C677T polymorphism and freshwater fish consumption on colon cancer risk (*P* value for interaction = 0.031). Interactions were observed between the *MTHFR* A1298C polymorphism and bowel habits, family history of cancer, alcohol consumption, and beef consumption on colon cancer risk (*P*-value for interaction = 0.0005, 0.007, 0.067, 0.003, respectively).

**Conclusions:**

In a Thai population, colon cancer risk was associated with alcohol and beef consumption, bowel habits, and family history of cancer. Interactions between *MTHFR* polymorphisms and environmental factors were also observed.

## INTRODUCTION

In Thailand, colorectal cancer is one of the 10 most common cancers, and incidence has been increasing in both sexes in all areas during 1988–2000.^1^^–^^4^ The latest estimated annual incidence rate of colorectal cancer in the Thai population (1999) was 8.8 and 7.6 per 100 000 population in males and females, respectively. The population-based cancer registry of Khon Kaen, a province in northeastern Thailand, showed that the incidence of colorectal cancer during 1998–2000 was 8.6 and 7.0 per 100 000 population in males and females, respectively.^1^^–^^4^ Of these incident cases, approximately 60% were colon cancers and 40% were rectal cancers. An increasing trend was observed for both.

A number of behavioral and environmental factors have been linked with colorectal cancer risk.^5^^–^^16^ Of these, obesity, physical inactivity, and alcohol consumption have been most consistently associated with increased risk of colon cancer. On the other hand, use of nonsteroidal anti-inflammatory drugs and high consumption of vegetables and fruits are generally associated with a decreased risk of colorectal cancer. The findings on smoking are not consistent. In epidemiological studies, diets low in folate have been found to increase the risk of colon cancer.^17^^–^^19^ Other dietary factors, including methionine and vitamins B_6_ and B_12_, have been associated with colon cancer in some but not all epidemiological studies.^7^^,^^11^^,^^19^^–^^21^ Methylenetetrahydrofolate reductase (MTHFR) is an important enzyme in folate metabolism; it catalyzes the conversion of 5,10-methylenetetrahydrofolate (5,10-methylene-THF) to 5-methyltetrahydrofolate (5-methyl-THF).^22^ Two common polymorphisms in the *MTHFR* gene have been characterized.^23^^,^^24^ C677T causes an alanine to valine substitution in the N-terminal catalytic domain, which reduces enzyme activity and leads to lower levels of circulating folate (5-methyl-THF), accumulation of 5,10-methylene-THF, and increased plasma homocysteine.^25^^,^^26^

This functional polymorphism has attracted a great deal of attention with regard to cancer risk, but the results have been conflicting. Several studies have shown that the low-activity variants of *MTHFR* C677T and A1298C are associated with decreased risks of colon cancer^27^^–^^29^ and acute lymphocytic leukemia.^30^ However, the same variants have also been linked with an increased risk of endometrial cancer,^31^ cervical intraepithelial neoplasia,^32^ esophageal squamous cell carcinoma,^33^ gastric cancer,^34^ and bladder cancer.^35^ To our knowledge, no studies of this topic have been conducted in Thailand.

As part of the multicenter “International collaborative epidemiological study of host and environmental factors for stomach and colorectal cancers in Southeast Asian Countries”, we examined putative risk factors for colon cancer in a population from northeastern Thailand, with a focus on both environmental parameters and polymorphisms in *MTHFR* C677T and A1298C.

## METHODS

### Subjects

A total of 130 new cases of colon cancer were recruited from Srinagarind Hospital and Khon Kaen Regional Hospital, Khon Kaen Province, between October 2002 and October 2006. All patients were from Khon Kaen Province or neighboring provinces and were histologically confirmed to have colon cancer. The patients were interviewed within 3 months of diagnosis. During the same period, 1 control matched for sex, age (±3 years), and province of residence was recruited for each case. Subjects with gastrointestinal disease or other cancers were excluded. All gave informed consent for their participation in the study. Subjects who refused to complete the interview or were unable to do so because of advanced age or other reasons were excluded. The controls had a variety of illnesses, the most common of which were inflammation, and diseases and disorders of the eye and genitourinary system. A 5-ml blood sample was obtained from all cases and their matched controls, and transferred to the laboratory for investigation of polymorphisms in the *MTHFR* gene.

### Interview

Subjects were interviewed by 2 trained interviewers using a structured questionnaire comprising 2 sections. The first section included demographic and socioeconomic status, smoking history, family history of cancer, past history of illness, and bowel habits. The second section was a food frequency questionnaire structured by meal. There were 9 categories of food items. The questions for each item consisted of frequency of consumption (daily, weekly, monthly, less than once a month) and amount consumed per unit of frequency. Support for the validity of this dietary method is provided by our previous study.^36^ For beverage consumption, there were 2 groups: alcoholic beverages and tea/coffee. The questions for each item requested information on whether the participant did or did not drink the beverage, the frequency of drinking, and the amount consumed per occasion.

The interview requested information on habits 1 year before the subjects became sick with their present illness. For bowel habits, the interviewers asked the subjects to recollect these habits beginning from adolescence to working age, until 1 year before the present illness, and asked the subjects to determine if their bowel habits had changed.

### Laboratory methods

Genomic DNA was extracted from buffy coat fractions of the cases and their matched controls using a standard technique at Nagoya City University Medical School, Nagoya, Japan. Gene amplification and polymorphism analyses were performed in the Microbiology Laboratory at the Faculty of Medicine, Khon Kaen University, Thailand.

**Figure 1.**
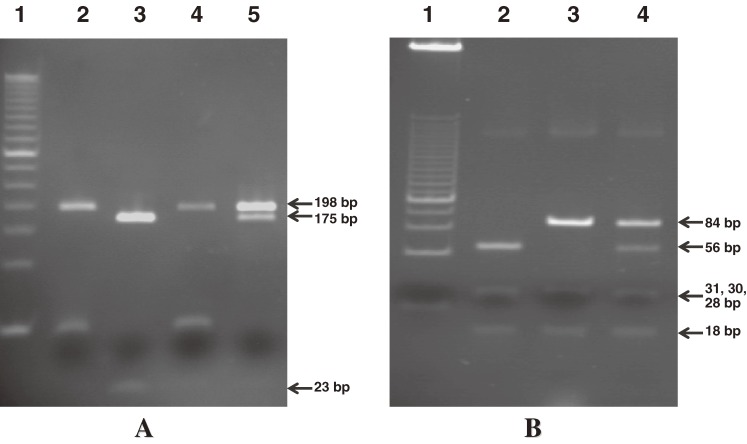
*MTHFR* polymorphism was analyzed by PCR-RFLP. (A) The polymorphism at position 677 was analyzed by PCR followed by *Hinf*I restriction enzyme digestion. Lane 1, marker 50 bp; lanes 2 and 4, wild-type homozygotes; lane 3, mutant homozygotes; lane 5, mutant heterozygotes. (B) The polymorphism at position 1298 was examined by PCR followed by *Mbo*II restriction enzyme digestion. Lane 1, marker 25 bp; lane 2, wild-type homozygotes; lane 3, mutant homozygotes; lane 4, mutant heterozygotes. PCR-RFLP, polymerase chain reaction with restriction fragment length polymorphism.

The polymerase chain reaction with restriction fragment length polymorphism (PCR-RFLP) technique was modified as previously described.^23^ Briefly, amplification of *MTHFR* C677T used 2 primers, [F]:5′-TGA AGG AGA AGG TGT CTG CGG GA-3′ and [R]:5′-AGG ACG GTG CGG TGA GAG TG-3′. The PCR product (198 bp) was digested with 10 units of *Hinf*I restriction enzyme (Fermentas Life Sciences) in a 20-µl reaction mixture containing 5 µl of PCR fragments and 2 µl of 10× buffer R at 37°C overnight. Digestion products were visualized after electrophoresis on 8% polyacrylamide with ethidium bromide. Results of the *Hinf*I RFLP analysis of the *MTHFR* C677T polymorphism are illustrated in Figure 1A . The C/C wild-type homozygote has only 1 (198 bp) fragment, the C/T heterozygote has 3 fragments (198, 175, and 23 bp), and the T/T mutant homozygote has 2 (175 and 23 bp) fragments.

Analysis of the *MTHFR* A1298C polymorphism was performed as previously described.^24^ The primers for PCR amplification were [F]:5′-AGG ACG GTG CGG TGA GAG TG-3′ and [R]:5′-CAC TTT GTG ACC ATT CCG GTT TG-3′. The PCR product of A1298C (163 bp) was digested with 10 units of *Mbo*II restriction enzyme (Fermentas Life Sciences) in a 20-µl reaction mixture containing 5 µl of PCR fragments and 2 µl of 10× buffer R at 37°C overnight. Digestion products were visualized after electrophoresis on 12% polyacrylamide with ethidium bromide. The *Mbo*II RFLP analysis for *MTHFR* A1298C polymorphism is illustrated in Figure 1B. The A/A wild-type homozygotes had 5 fragments (56, 31, 30, 28, and 18 bp), the A/C heterozygotes had 6 fragments (84, 56, 31, 30, 28, and 18 bp), and the C/C mutant homozygotes had 4 fragments (84, 31, 30, and 18 bp).

### Statistical analysis

Associations between colon cancer and potential risk factors were evaluated using odds ratios (ORs) and 95% confidence intervals (95% CIs) derived from conditional logistic regression. Crude and adjusted odds ratios were estimated for each independent variable. Factors included in the multivariate analysis by conditional logistic regression were those found to be strongly associated with colon cancer in univariate analysis and those that were not found to be strongly associated with colon cancer in univariate analysis but were shown in previous reports to play an important role in colon cancer risk. Possible modifications of the effects of potential risk factors by polymorphisms in *MTHFR* C677T and *MTHFR* A1298C were also analyzed.

The distributions of alleles/genotypes for the *MTHFR* C677T and A1298C polymorphisms were analyzed in the colon cancer cases and their matched controls by using the McNemar test; 2 × 2 tables were employed to compare genotype distributions between any 2 groups. Both tests were utilized to compare the cases and their matched control subjects with regard to genotype frequencies and potential risk factors for colon cancer such as demographic characteristics, diet, smoking, and alcohol drinking.

To investigate the gene–environment and lifestyle interactions, ORs and their 95% CI were calculated using the McNemar test. The ORs were adjusted for age and sex according to the *MTHFR* C677T and *MTHFR* A1298C polymorphisms. All statistical analyses were performed using STATA version 10, and a *P* value <0.05 was considered statistically significant.

For the analysis of cigarette smoking, the subjects were categorized as smokers or nonsmokers. Smokers included those who smoked filtered cigarettes, unfiltered cigarettes, or *yamuan* (homemade cigarette-sized cigars). Duration of smoking and average number of cigarettes per year were computed based on all smoking periods reported, and patients were dichotomized using the median for the controls. Average number of cigarettes was calculated as annual cigarette consumption (filtered and unfiltered) plus 1.5 times annual *yamuan* consumption. The 1.5 correction factor was used because *yamuan* are larger than regular cigarettes. The amount was categorized based on the 50th percentile of the controls and dichotomized into low and high levels.

For the analysis of alcohol drinking, there were 2 categories: drinkers and nondrinkers. Ever drinkers were defined as those who consumed at least 1 type of alcoholic beverage (beer, Thai rice wine [*sato*], white whiskey, Thai and other whiskies) at least once a month. Those who did not drink, or consumed alcoholic beverages less than once a month, were categorized as nondrinkers. The alcohol consumption of each subject was calculated as alcohol units. A unit of alcohol was defined as 10 milliliters (or approximately 8 grams) of ethyl alcohol. The number of units of alcohol in a drink was determined by multiplying the volume of the drink (in milliliters) by its alcohol percentage, and dividing by 1000.^37^

The average amount of alcohol consumed was analyzed based on grams per day, with the unit of alcohol consumption measure and percentage of alcohol by volume (%alc/vol) defined as 5.0% for beer, 7.0% for *sato*, 40% for white whisky, and 35% for other whiskies. The averages were calculated, converted into units of alcohol per day, and divided into 3 categories: nondrinker, ≤0.50 units per day, and >0.50 units per day.

For the analyses of dietary intake within the previous year (vegetables, fruits, freshwater and saltwater fish/shellfish, beef, and fried beef), 2 levels were used—low and high. Frequencies of each dietary intake, and the amount of intake per year, were computed based on each type of dietary intake reported. Patients were then dichotomized using the median for the controls.

## RESULTS

Table 1 shows the distribution of general characteristics in cases and controls. Because this was a matched case–control study, the distributions of age, sex, and province of residence were the same in cases and controls. Most subjects were laborers engaged in agricultural work. The median monthly household income for both cases and controls was similar (3000 Baht). In the cases, the primary tumor site was the sigmoid colon in 31.5%, the ascending colon in 8.5%, the cecum in 6.1%, the descending colon in 5.4%, the transverse colon in 3.9%, the hepatic flexure of the colon in 2.3%, the splenic flexure of the colon in 2.3%, and the overlapping region of the rectum in 0.8%. The remaining 39.2% were classified simply as “colon cancer.” Overall, 93.8% of the cancers were adenocarcinomas.

**Table 1.** Characteristics of cases and controls

Variables	Cases		Controls	
	
	*n* = 130	%	*n* = 130	%
Sex				
Male	71	54.6	71	54.6
Female	59	45.4	59	45.4
Age (years)				
≤40	26	20	24	18.5
41–50	28	21.5	28	21.5
51–60	44	33.9	44	33.8
>60	32	24.6	34	26.2
Mean (SD)	51.9 (11.8)		51.8 (11.9)	
Median (min:max)	53 (16:79)		53 (18:76)	
Marital status				
Single	3	2.3	12	9.2
Married	113	86.9	99	76.2
Separated, widowed	14	10.8	19	14.6
Occupation				
Agriculture, farmer	88	68.2	79	61.7
Office work, technical work	14	10.9	15	11.7
Professional work	12	9.3	19	14.9
Others	15	11.6	15	11.7
Education				
Illiterate	4	3.1	4	3.1
Primary school	98	75.4	96	73.8
Secondary school or higher	28	21.5	30	23.1
Household income per year (Baht)				
≤20 000	39	30	39	30
20 001–40 000	39	30	37	28.5
40 001–60 000	12	9.2	13	10
>60 000	40	30.8	41	31.5
Median (min:max)	36 000 (6000:720 000)		36 000 (4992:960 000)	
*MTHFR* C677T polymorphism				
C/C	104	80	94	72.3
C/T	26	20	31	23.8
T/T	0	0	5	3.9
*MTHFR* A1298C polymorphism				
A/A	43	33.1	54	41.5
A/C	84	64.6	71	54.6
C/C	3	2.3	5	3.9

Among the cases (130) and controls (130) genotyped for the *MTHFR* C677T polymorphism, the prevalence of the T allele was 20% and 27.7%, respectively. For the *MTHFR* A1298C polymorphism, the frequency of the C allele was 66.9% in cases and 58.5% in controls (Table 1).

Table 2 shows the results of univariate analysis. There was no significant association between genotype and colon cancer risk. As compared with subjects having the *MTHFR* 677 C/C genotype, those with the *MTHFR* 677 C/T genotype had a tendency toward reduced risk (OR = 0.72; 95% CI: 0.39–1.32). Those with the *MTHFR* 1298 A/C genotype had a tendency toward higher risk of colon cancer than did those with the 1298 A/A genotype (1.50; 0.89–2.53), whereas those with the *MTHFR* 1298 C/C genotype had a tendency for decreased risk, as compared with patients with the 1298 A/A genotype (0.65; 0.11–3.70).

**Table 2.** Univariate analysis of potential risk factors for colon cancer

Variables	Cases		Controls		OR^a^	95% CI	*P*-value
	
	*n*	%	*n*	%			
Bowel habits							
Normal	51	39.8	89	70.6	1		
Occasional constipation	62	48.5	34	27	1.43	1.83–6.43	<0.001
Frequent constipation	15	11.7	3	2.4	10.67	2.29–49.75	0.003
Family history of cancer							
No	79	61.2	97	75.2	1		
Yes	50	38.8	32	24.8	1.95	1.11–3.53	0.013
Occupation							
Agriculture, farmer	88	68.2	79	61.7	1		
Office work, technical work	14	10.9	15	11.7	0.91	0.44–1.89	0.804
Professional work	12	9.3	19	14.9	0.6	0.28–1.27	0.183
Others	15	11.6	15	11.7	0.84	0.35–2.00	0.696
Smoking							
No	70	53.8	72	55.4	1		
Yes	60	46.2	58	44.6	1.18	0.49–2.91	0.683
Average no. of cigarettes per year							
Nonsmoker	70	54.3	72	55.4	1		
Low (1–3650)	40	31	39	30	1.21	0.52–2.82	0.666
High (>3650)	19	14.7	19	14.6	1.14	0.45–2.88	0.776
Alcohol drinking							
No	59	45.4	66	50.8	1		
Yes	71	54.6	64	49.2	1.47	0.73–3.04	0.25
Frequency of alcohol consumption							
Nondrinker	59	45.4	66	50.8	1		
<1/month	26	20	28	21.5	1.21	0.56–2.59	0.625
Weekly	22	16.9	15	11.5	1.92	0.82–4.49	0.134
Daily	23	17.7	21	16.2	1.55	0.65–3.66	0.321
Units of alcohol per day							
Nondrinker	73	56.5	84	64.6	1		
≤0.50	21	16.1	13	10	2.15	0.94–4.95	0.071
>0.50	36	27.7	33	25.4	1.54	0.77–3.08	0.226
Tea or coffee drinking							
No	71	54.6	79	60.8	1		
Yes	59	45.4	51	39.2	1.33	0.76–2.36	0.285
Beef (average times/day)							
Low (≤0.08; or ≤2.4 times per month)	83	63.8	97	74.6	1		
High (>0.08; or >2.4 times per month)	47	36.2	33	25.4	1.78	0.97–3.36	0.048
Pork (average times/day)							
Low (≤0.5)	90	69.2	92	70.8	1		
High (>0.5)	40	30.8	38	29.2	1.07	0.62–1.84	0.796
Poultry (average times/day)							
Low (≤0.2)	99	76.2	108	83.1	1		
High (>0.2)	31	23.8	22	16.9	1.53	0.80–3.00	0.17
Freshwater fish (average times/day)							
Low (≤1)	124	95.4	116	89.2	1		
High (>1)	6	4.6	14	10.8	0.38	0.11–1.15	0.059
Saltwater fish and shellfish (average times/day)							
Low (≤0.23)	87	66.9	97	74.6	1		
High (>0.23)	43	33.1	33	25.4	1.59	0.83–3.11	0.132
Offal (average times/day)							
Low (≤0.2)	116	89.9	123	94.6	1		
High (>0.2)	14	10.1	7	5.4	2.4	0.79–8.69	0.089
Vegetables (average times/day)							
Low (≤2.0)	93	71.5	90	69.2	1		
High (>2.0)	37	28.5	40	30.8	0.88	0.48–1.61	0.668
Fruits (average times/day)							
Low (≤3.51)	103	79.2	97	74.6	1		
High (>3.51)	27	20.8	33	25.4	0.77	0.41–1.43	0.376
Salt							
Rock salt	67	53.6	71	58.2	1		
Sea salt	21	16.8	16	13.1	1.27	0.59–2.76	0.538
Both	37	29.6	35	28.7	1.09	0.58–2.07	0.786
*MTHFR* C677T polymorphism							
C/C	104	80	94	72.3	1		
C/T	26	20	31	23.9	0.72	0.39–1.32	0.288
T/T	0	0	5	3.8	—		
*MTHFR* A1298C polymorphism							
A/A	43	33.1	54	41.5	1		
A/C	84	64.6	71	54.6	1.5	0.89–2.53	0.129
C/C	3	2.3	5	3.9	0.65	0.11–3.70	0.629

^a^Crude odds ratio from matched case–control analysis.

There were no associations between colon cancer risk and occupation, smoking status, or tea/coffee drinking. There was an association between alcohol consumption and colon cancer, as shown in Table 2. Those with a family history of cancer had a higher risk of colon cancer than did those without such a family history (1.95; 1.11–3.53). Those who reported occasional or frequent constipation had significantly elevated risks of colon cancer (1.43; 1.83–6.43, and 10.67; 2.29–49.75, respectively), as compared with those who reported normal bowel habits.

On univariate analyses of dietary intake based on the food frequency questionnaire, with the low level as the referent group, there was no significant association between overall food consumption and colon cancer risk. Only beef consumption showed a possible link with colon cancer risk (1.78; 0.97–3.36).

Factors found to have a strong association with colon cancer on univariate analysis (a *P* value <0.10), and factors that have been reported to have a strong relationship with colon cancer, were included in the multivariate analysis by conditional logistic regression. These factors were bowel habits; family history of cancer; alcohol consumed per day; and consumption of beef, freshwater fish, offal, and fruit.

Table 3 shows the adjusted ORs and 95% CIs from the multivariate analysis. The factors that remained as significant risk factors were bowel habits and alcohol consumption. Patients with occasional or frequent constipation had significantly higher risks for colon cancer than did those with normal bowel habits (OR = 3.43; 95% CI: 1.72–6.82, and OR = 11.69; 95% CI: 2.18–62.79, respectively). Alcohol drinkers who consumed ≤0.50 units of alcohol per day had a significantly higher risk than did nondrinkers (3.50; 1.19–10.25). Drinkers who consumed >0.50 units of alcohol per day also had a higher risk than nondrinkers, but the difference was not statistically significant (1.71; 0.74–3.96).

**Table 3.** Multivariate analysis of potential risk factors for colon cancer

Variables	OR^a^	OR^b^	95% CI	*P*-value
Bowel habits				
Normal	1	1		
Occasional constipation	1.43	3.43	1.72–6.82	<0.001
Frequent constipation	10.67	11.69	2.18–62.79	0.004
Family history of cancer				
No	1	1		
Yes	1.95	1.32	0.68–2.54	0.414
Units of alcohol per day				
Nondrinker	1	1		
≤0.50	2.15	3.5	1.19–10.25	0.023
>0.50	1.54	1.71	0.74–3.96	0.208
Beef (average times/day)				
Low (≤0.08; or ≤2.4 times per month)	1	1		
High (>0.08; or >2.4 times per month)	1.78	1.56	0.74–3.28	0.238
Offal (average times/day)				
Low (≤0.2)	1	1		
High (>0.2)	2.4	1.89	0.59–6.03	0.284
Freshwater fish (average times/day)				
Low (≤1)	1	1		
High (>1)	0.38	0.62	0.18–2.16	0.451
Fruit (average times/day)				
Low (≤3.51)	1	1		
High (>3.51)	0.77	0.64	0.29–1.37	0.25

^a^Crude odds ratio, ^b^Adjusted odds ratio.

Risk factors that were identified as significant were studied for interaction with polymorphisms in *MTHFR* C677T and A1298C with regard to the risk for colon cancer. The results are shown in Table 4. There was an interaction between freshwater fish consumption and polymorphism in *MTHFR* C677T (*P* value for interaction = 0.031). For the *MTHFR* A1298C polymorphism, there were interactions with bowel habits, family history of cancer, and beef consumption (*P* value for interaction = 0.0005, 0.007, and 0.003, respectively).

**Table 4.** Gene–environment interactions of *MTHFR* C677T and A1298C polymorphisms with potential risk factors for colon cancer

*MTHFR*	Environment/lifestyle factors	Cases (*n*)	Controls (*n*)	Adjusted OR (95% CI)	*P*-value	*P*-value*
C677T	Freshwater fish (times/day)					0.031
C/C	Low	98	85	1		
C/C	High	6	9	0.68 (0.17–2.71)	0.58	
C/T	Low	26	27	0.97 (0.44–2.12)	0.936	
C/T	High	0	4	—	—	
T/T	Low	0	4	—	—	
T/T	High	0	1	—	—	
A1298C	Bowel habits					0.0005
A/A	Normal	18	38	1		
A/A	Occasional constipation	22	13	3.9 (1.47–10.37)	0.006	
A/A	Frequent constipation	3	1	8.57 (0.43–171.5)	0.16	
A/C	Normal	32	47	1.4 (0.60–3.29)	0.438	
A/C	Occasional constipation	40	20	4.53 (1.71–12.03)	0.002	
A/C	Frequent constipation	10	2	12.28 (1.49–101.41)	0.02	
C/C	Normal	1	4	2.59 (0.12–54.42)	0.543	
C/C	Occasional constipation	0	1	—	—	
C/C	Frequent constipation	2	0	—	—	
A1298C	Family history of cancer					0.007
A/A	No	30	37	1		
A/A	Yes	13	16	1.03 (0.33–3.21)	0.955	
A/C	No	47	56	1.01 (0.45–2.24)	0.994	
A/C	Yes	36	15	2.01 (0.81–5.03)	0.132	
C/C	No	2	4	1.96 (0.18–21.15)	0.581	
C/C	Yes	1	1	0.99 (0.02–49.42)	0.997	
A1298C	Units of alcohol per day					0.067
A/A	Nondrinker	26	35	1		
A/A	≤0.50	5	5	1.84 (0.38–8.85)	0.445	
A/A	>0.50	12	14	1.93 (0.59–6.25)	0.276	
A/C	Nondrinker	46	46	1.24 (0.57–2.74)	0.587	
A/C	≤0.50	14	7	5.88 (1.21–28.47)	0.028	
A/C	>0.50	24	18	2.12 (0.64–7.02)	0.219	
C/C	Nondrinker	1	3			
C/C	≤0.50	2	1	17.19 (0.34–863.6)	0.155	
C/C	>0.50	0	1	—	—	
A1298C	Beef consumption					0.003
A/A	Low	33	38	1		
A/A	High	10	16	0.93 (0.32–2.71)	0.898	
A/C	Low	48	55	1.14 (0.56–2.32)	0.717	
A/C	High	36	16	2.07 (0.79–5.42)	0.137	
C/C	Low	2	4	1.24 (0.09–17.05)	0.874	
C/C	High	1	1	—	—	

*P*-value* = *P*-value for interaction.

## DISCUSSION

In northeastern Thailand, colon cancer is one of the 10 most common cancers and the incidence rate is increasing,^4^ although absolute values are much lower than those in Japan and European countries.^38^ In the present study, the authors recruited cases from 2 hospitals: Srinagarind Hospital, which is a teaching hospital, and Khon Kaen Regional Hospital, which specializes in cancer treatment. A great number of cancer patients come to these 2 hospitals for treatment. Therefore, we were able to recruit a sufficient number of cases for this study.

Our present results augment earlier findings on lifestyle-related risk factors for colorectal cancer in northeastern Thailand.^10^ The strongest association in the present study was between constipation and colon cancer. Sonnenberg and Muller^39^ conducted a systematic review of 9 case–control studies published before 1992, and evaluated the risk for colorectal cancer associated with self-reported constipation and bowel movement frequency: constipation was associated with a significant increase in the risk of colorectal cancer (OR, 1.48; 95% CI, 1.32–1.66). Although we only investigated colon cancer, our findings are consonant with those of earlier studies. It has been hypothesized that constipation increases colon cancer risk because it results in longer transit times in the colon, which increases the duration of contact between the colonic mucosa and concentrated carcinogens in the lumen.^40^^,^^41^ Possible carcinogenic agents within stool include bile acids,^42^^,^^43^ fecapentaene,^44^ and ammonium acetate.^45^ Constipation is also a major symptom of colorectal cancer; however, in this study the authors informed participants that they should focus on bowel habits that began in adolescence through working age until 1 year before illness, and reminded them to think of any changes in bowel habits, to avoid information bias.

A number of studies have found an association between beef consumption and the risk of colorectal cancer.^6^ Important sources of known heterocyclic amine carcinogens with established links to colorectal cancer^46^ include cooked meat (which contains heterocyclic amines and nitrosamines). In our study, there was a trend toward increasing risk of colon cancer with high beef consumption in both univariate and multivariate analyses, but the associations were not significant. However, in the study of possible gene–environment interactions between the *MTHFR* A1298C polymorphism and beef consumption, an interaction was observed (*P* value for interaction = 0.003). This finding highlights an important public health concern, especially for those who eat beef.

An analysis of cancer registries in Thailand shows that most patients present at a late stage,^1^^–^^4^^,^^47^ which might be partly due to the lack of effective cancer screening in Thailand. Northeastern Thailand has, for many decades, been the most impoverished part of the country. Until the introduction of various industries in the last 20 years, the population was largely rural, relying on cultivation of rice as the staple food. The genetic background of this region has not changed, so the increase in the incidence of colon cancer must be due to lifestyle or environment changes.

A number of studies have reported that high alcohol consumption increases the risk of colon cancer and that the association is likely dose-dependent, irrespective of the type of alcohol consumed.^5^^,^^12^^,^^13^ Ethanol may act as a solvent or have cytotoxic effects on tissues. It may also cause deficiencies in nutrients, particularly folate.^7^^,^^48^ In this study, there was a trend toward an association between alcohol consumption and the risk of colon cancer, with a possible link to the *MTHFR* A1298C polymorphism.

Several epidemiological studies have provided evidence that high consumption of vegetables and fruits lowers the risk of colon cancer.^6^^,^^49^ Although our results showed only a tendency for protection against colon cancer, vegetables and fruits possess antioxidants that can reduce DNA damage.^46^ In addition, a key constituent of vegetables and fruits is folate. There is evidence that consumption of folate is related to the risk of adenomas and other types of neoplasms. Folate and *MTHFR* may be important in influencing the availability of S-adenosylmethionine (SAM), the universal methyl donor, thereby affecting both DNA methylation and the nucleotide pool.^24^ A deficiency of folate in tissues with rapidly replicating cells results in ineffective DNA synthesis, which reduces cell proliferation, impairs cellular physiology, and alters cell morphology. The MTHFR enzyme plays a central role in the metabolism of folate, a nutrient that has been inversely associated with colorectal cancer risk.

Several reports have shown that the *MTHFR* C677T polymorphism is associated with a reduced risk of colorectal cancer, especially in people who consume high levels of folate^27^^,^^29^^,^^50^^–^^55^ or take multivitamin supplements that include B_2_, B_6_, and B_12_,^29^^,^^56^ but an increased risk has been noted in those who consume a large amount of alcohol. The importance of folate was confirmed by an in vitro study of HCT116 cells, in which the *MTHFR* C677T mutation was associated with significantly increased genomic DNA methylation when the folate supply was adequate or high. However, in cases of folate insufficiency, the mutation was associated with significantly decreased genomic DNA methylation.^57^

In this present study, the genotype frequency of *MTHFR* 677 was 27.7% in controls, which corresponds to other studies.^27^^,^^50^^–^^54^^,^^56^^,^^58^^,^^59^ This polymorphism has been shown to modify the risk for several cancers in a site-specific manner.^60^^,^^61^ Epidemiologic evidence indicates that this polymorphism has a protective effect in individuals with adequate or high levels of folate and other nutrients involved in 1-carbon metabolism.^62^^–^^64^ In those with inadequate levels of folate and related nutrients, the protective effect conferred by this polymorphism appears to be lower; in some cases, an increased risk of colorectal cancer has been observed.^64^

In our analysis of the *MTHFR* 1298 polymorphism, the genotype distribution in the controls deviated from the Hardy–Weinberg equilibrium. The rate for the A1298C polymorphism in controls was higher than rates noted in reports from other countries. Our figures may be representative for the Thai population; however, there have been no previous reports. The possibility of genotyping error should be acknowledged. Genotyping error in *MTHFR* 1298 polymorphism studies has been reported when genotyping was performed by digesting PCR products with the enzyme *Mbo*ll, which recognizes the sequence GAAGAN8 and digests this sequence on wild allele 1298A (A/A). If both 1298A and 1317C are present on a chromosome, the fragment size of digestion does not differ and it is thus difficult to differentiate heterozygous individuals (1298 A/C genotype) from wild homozygous individuals (1298 A/A genotype), even when a positive control is included in all the electrophoresis runs to compare the sample results.^65^^,^^66^ Therefore, fragment size analysis by sequencing and comparison of the sequence in GenBank is necessary to confirm the result. In a comparison of RFLP using the enzyme *Mbo*II and direct sequencing techniques for identification of polymorphism in A1298C, an 8.6% difference in genotyping was found.^67^

As is the case with any case–control study, the possibility that cases and controls differentially reported inaccurate information should be considered when interpreting the results. A differential reluctance on the part of cases or controls to accurately report average levels of drinking may have occurred in this study. One might expect the underestimation of true alcohol drinking to have occurred at some level above 1 drink per day (arguably, a socially acceptable level in Thailand), which would result in inconsistency in the association of colon cancer with the level of alcohol consumption.

Associations of various types of cancer with food groups and nutrient compositions have been studied elsewhere. Although we made great efforts to use available investigative tools in this study, there is no standardized food frequency questionnaire for northeastern Thai food. Thai eating habits and foods are quite different from those in the West, so it is difficult at present to quantify foods and calculate the precise intakes of vitamins and nutrients.

One other limitation in this study is that its relatively small size probably had some impact on the analysis of interactions, as evidenced by the absence of data for some variables in the tables. A future study with a larger sample size is warranted.

### Conclusion and recommendations

We conclude that bowel habits and family history of cancer are strongly associated with the risk of colon cancer in this population. The findings also suggest that individuals who consume alcohol and beef are at increased risk. Interactions of lifestyle factors with *MTHFR* C677T and *MTHFR* A1298C polymorphisms were found but require additional investigation. Therefore, further research on the mechanisms underlying carcinogenesis in the Thai population is required.
